# Analysis on anatomical references to assess the coronal alignment of tibial and femoral cuts in mega prosthetic knee replacement

**DOI:** 10.1007/s10195-013-0277-4

**Published:** 2013-12-21

**Authors:** Vikas Karade, Bhallamudi Ravi

**Affiliations:** Department of Mechanical Engineering, Indian Institute of Technology Bombay, Mumbai, 400076 Maharashtra India

**Keywords:** Knee replacement, Anatomical reference, Implant, Cutting alignment, Cutting guide

## Abstract

**Background:**

In megaprosthetic knee replacement, surgeons use cutting guides that depend on anatomLevel of evidence

ical references to determine the ideal cutting plane alignment. In this work, we investigated the accuracy of using femoral cortical surfaces and tibial canal portions as the references. The study aims to improve the design and use of the cutting guides.

**Materials and methods:**

Sixty-one knee scanograms of 33 patients (mean age around 20 years) diagnosed with osteogenic sarcoma and undergoing distal femur megaprosthetic surgery were acquired. Angles between the selected anatomical references and axis perpendicular to the ideal cutting plane (anatomical axis for femur and mechanical axis for tibia) were measured for both femur and tibia, in coronal view. The smaller the magnitude of the angles, the better the anatomical reference is.

**Results:**

At the central femoral region, on average, both lateral and medial cortical surfaces give accurate alignment of the ideal cutting plane (0.6° and 0.8°, respectively), with no significant difference (*p* > 0.01). At the distal region, the lateral cortical surface gives significantly better alignment compared to the medial cortical surface (*p* < 0.01), but not as accurate (1.4°) as in the central region. For tibia, the central tibial canal gives significantly accurate alignment of the ideal cutting plane (−0.3°) on average, compared to the proximal tibial canal (*p* < 0.01).

**Conclusions:**

For a femoral cut, both lateral and medial cortical surfaces are the best anatomical references, but only at the central region. For a tibial cut, the central anatomical axis is the best reference.

**Level of evidence:**

IV.

## Introduction

**Fig. 1:**
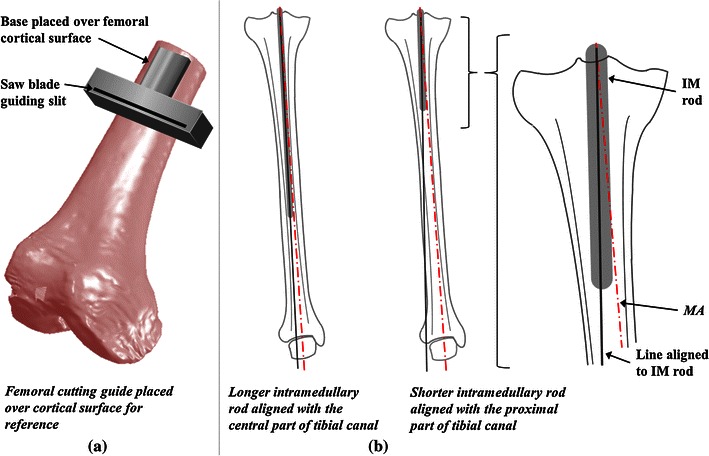
Anatomical references for femoral and tibial cuts: **a** femoral cutting guide **b** different lengths of intramedullary rods used with tibial cutting guide

In megaprosthetic knee replacement, a femoral component placed over the cut surface of the femoral shaft articulates with a tibial component placed over the cut surface of the tibial plateau [12]. The alignment of the femoral and tibial components in the coronal plane is one of the most important factors for the success of the surgery [3, 6]. In the coronal plane, the ideal femoral cutting plane is perpendicular to its anatomical axis and the ideal tibial cutting plane is perpendicular to its mechanical axis (MA) [3, 4, 7]. Deviation of the cut surface from the ideal cutting plane alignment can lead to malpositioning of the components and hence an undesirable load distribution, resulting in loosening, and ultimately failure of the surgery [7]. Specially designed cutting guides use anatomical references to determine the ideal cutting plane alignment [11, 15]. For a femoral cut, a cutting guide as shown in Fig. 1a uses the outer cortical surface (medial or lateral) as a reference to determine the alignment of femoral anatomical axis (FAA), and hence the ideal cutting plane. However, there are no scientific anatomical studies to identify the anatomical reference that gives accurate alignment for distal femur megaprosthetic surgery. Similarly for a tibial cut, intramedullary cutting guides refer to the tibial canal using intramedullary rod to determine the alignment of tibial MA, and hence the ideal cutting plane [8]. Here it is assumed that the tibial canal axis is parallel to the MA of the tibia [11, 13, 14]. It has, however, not been shown which part of the canal (proximal or central) gives the alignment of the MA more accurately in the coronal plane (Fig. 1b).

For both femoral and tibial cuts, it is unclear which anatomical reference, when being referred by the cutting guides, will result in more accurate bone cuts. Therefore, we investigated and compared the accuracy of using femoral cortical surfaces (medial and lateral) and tibial canal portions (proximal and central) as references to assess the alignment of the ideal cuts in the coronal plane, for megaprosthetic knee replacement.

## Materials and methods

The study was performed on randomly accessed medical preoperative scanograms (in the coronal view) of Indian patients diagnosed with osteogenic sarcoma, undergoing distal femur megaprosthetic knee replacement surgery. In the femoral study, 61 knees in scanograms of 33 patients were used. This study group consisted of 19 males (mean age 18.6 years; range 11–50 years) and 14 females (mean age 20.6 years; range 13–40 years). In the tibial study, 59 knees in scanograms of 30 patients were used. This study group consisted of 17 males (mean age 21.4 years; range 11–50 years) and 13 females (mean age 17.2 years; range 13–40 years).

**Fig. 2:**
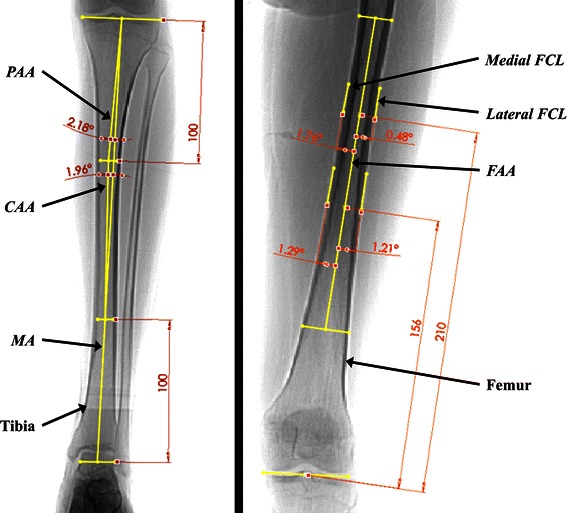
Measurement of angles between selected anatomical references and cutting axis

In the femoral study, lateral and medial cortical surfaces of femur were selected as anatomical references to determine the alignment of the ideal femoral cut. We defined lateral and medial femoral cortical lines (FCLs) (lateral FCL and medial FCL) as the lines representing the lateral and medial surface of the femur, respectively, on coronal view scanogram (Fig. 2). To measure the accuracy of using FCLs as the references, angles between FCLs and FAA were measured, because FAA is perpendicular to the ideal femoral cutting plane. Usually, the cutting guides are designed to access an axis (anatomical axis in the case of femur), and to guide a cut perpendicular to it. On the scanograms, FAA was drawn along the middle of the bone structure [11]. The lateral FCL and medial FCL were drawn at the edge of the femoral shaft on lateral and medial sides, respectively. Angles between FCLs and FAA were measured in all the scanograms. A positive value of the angle between medial or lateral FCL and FAA suggests that the FCL is moving toward medial or lateral side, respectively, as it starts from the proximal to distal region of the femur. These anatomical references were studied at two locations: at the central and the distal part of femur (Fig. 2). These locations were selected on the basis of standard lengths of the megaprosthetic femoral implant, because the cut is made at a position based on the implant length.

For the tibial study, the use of proximal anatomical axis (PAA) and central anatomical axis (CAA) of the tibia as the anatomical references to determine the alignment of tibial MA in coronal plane was investigated. PAA and CAA represent the axes of proximal and central part of the tibial canal, and are usually accessed by a shorter 10-cm and longer 18-cm intramedullary rod, respectively, while using intramedullary cutting guides [5]. The cuts are then made perpendicular to the intramedullary rod. To measure the accuracy of using PAA and CAA as the references, angles between them and tibial MA were measured, because MA is perpendicular to the ideal tibial cutting plane. On the scanograms, MA was drawn as a line through the center of knee and the center of ankle [11]. The PAA was drawn as a line connecting the center of the knee joint and the midpoint of inner cortical diameter at a location 10 cm distal to the knee joint in the coronal plane. The CAA was drawn as a line joining the midpoints of inner cortical diameter at a location 10 cm distal to the knee joint and at a location 10 cm proximal to the ankle joint. The angles between MA and PAA, as well as CAA and MA, were measured in all the scanograms (Fig. 2). The positive value of the angle PAA–MA suggests that the PAA is moving toward the lateral side of MA, and vice versa. The positive value of CAA–MA suggests that CAA is moving away from MA, as it starts from the proximal region to the distal region of the tibia (Table 1).

**Table 1:** Baseline patient characteristics

	Femoral study		Tibial study	
Number of knees	61		59	
Number of patients	33		30	
	Male	Female	Male	Female
Gender	19	14	17	13
Mean age, SD (years)	18.6, 8.5	20.6, 7.5	21.4, 10.6	17.2, 3.3
Median age (years)	17	18	18	17
Range of age (years)	11–50	13–40	11–50	13–40

All the scanograms were imported into CAD software (SolidWorks 2009) and lines corresponding to the cutting axis and selected anatomical references were drawn. Angular measurements were performed using the same software. The smaller the magnitude of the angles, the better the corresponding anatomical references represent the alignment of the corresponding ideal cutting planes. Table 2 summarizes the selected anatomical references for femur and tibia.

**Table 2:** Anatomical references selected and measurements performed in the study

Study	Angles measured between
Femur	Medial femoral cortical line (medial FCL) and femoral anatomical axis (FAA)
	Lateral femoral cortical line (lateral FCL) and femoral anatomical axis (FAA)
Tibia	Proximal anatomical axis (PAA) and mechanical axis (MA)
	Central anatomical axis (CAA) and mechanical axis (MA)

For all the parameters, values of the mean, standard deviation, median, and interquartile range were calculated. Three null hypotheses were stated: (a) there is no difference in the accuracy of using lateral and medial cortical surfaces as anatomical references to determine the ideal femoral cut at distal region of femur; (b) there is no difference in the accuracy of using lateral and medial cortical surfaces as anatomical reference to determine the ideal femoral cut at central region of femur; and (c) there is no difference in the accuracy of using proximal and central portion of tibial canal as anatomical reference to determine the ideal tibial cut. The angles between the axis perpendicular to the ideal cut (FAA in femur and MA in tibia) and various anatomical references (medial/lateral FCLs for femur and CAA/PAA for tibia) were compared by the Wilcoxon matched-pairs signed-rank test. The two-sided level of significance was kept as 0.01. The mathematical calculations and the statistical analysis were performed using Microsoft Excel 2007.

## Results

The values of the mean, standard deviation, median, and interquartile range of the angular measurements for both the studies are summarized in Table 3. In the femoral study, at the distance of 14 cm (i.e., distal region), the alignment of the lateral FCL was significantly closer (parallel) to that of the FAA compared to the alignment of the medial FCL (*p* < 0.01). Therefore we reject the first null hypothesis. The mean value of angle between the lateral FCL and FAA was 1.4° (SD = 2.0). Similarly, the angle between the medial FCL and FAA was 2.7° (SD = 1.6). At the distance of 21 cm (i.e., central region), the alignment of the lateral FCL and that of the medical FCL with respect to FAA was not significantly different (*p* > 0.01). Therefore, we fail to reject the second null hypothesis. The mean value of the angle between lateral FCL and FAA was 0.6° (SD = 1.5). Similarly, the angle between medial FCL and FAA was 0.8° (SD = 1.4). In the tibial study, the alignment of the CAA (central anatomical axis) was significantly closer (parallel) to that of the MA compared to the alignment of the PAA (*p* < 0.01). Therefore, we reject the first null hypothesis. The PAA–MA angle had the mean value of 0.7° (SD = 1.3). The CAA–MA angle had the mean value of −0.3° (SD = 0.7).

**Table 3:** Measurements of angles between selected anatomical references and axis perpendicular to the ideal cutting plane

Study	Lateral FCL–FAA mean (SD)		Medial FCL–FAA mean (SD)		Significance *p* value
Statistic	Mean (SD)	Median (IQR)	Mean (SD)	Median (IQR)	
Femoral anatomical references					
Distal portion (14 cm from distal end)	1.4 (2.0)	1.0 (0–2.1)	2.7 (1.6)	2.6 (1.7–3.9)	0.0005
Central portion (21 cm from distal end)	0.6 (1.5)	0.6 (−0.4 to 1.4	0.8 (1.4)	0.8 (0 to 1.5)	0.2501

Study	CAA–MA mean (SD)		PAA–MA mean (SD)		Significance *p* value
Statistic	Mean (SD)	Median (IQR)	Mean (SD)	Median (IQR)	
Tibial anatomical references					
	−0.3 (0.7)	0.8 (−0.7 to 0.3)	0.7 (1.3)	−0.2 (−0.4 to 1.7)	<0.0001

*SD* standard deviation*, IQR* inter-quartile region

## Discussion

Lateral and medial FCLs were chosen as anatomical references in this study, because using the bone surface directly as a reference and cutting the bone perpendicular to it would be the easiest approach for cutting guide design. The results show that at the central region of the femur, both the cortical surfaces (medial and lateral) can be accurate references (no significant difference, *p* > 0.01). At the central region, the mean angle between FCL and FAA was < 1° with standard deviation of around 1.5°. At the distal region, though the alignment of the lateral FCL is closer to that of FAA compared to the medial FCL (*p* < 0.01), the mean angle between them is more than 1° with a large standard deviation of 2°. Hence, both lateral and medial cortical surfaces cannot be used as the reference for accurate cut at distal femoral region.

For tibia, Yoo et al. [18] had shown that in the sagittal plane, the PAA is nearly parallel to the MA, and suggested using the PAA as a sagittal plane reference with intramedullary cutting guide. In our work, anatomical references were studied for the coronal plane alignment. Both proximal and central portion of the tibial canal were selected as the references. These references can be accessed by the intramedullary rod, depending on its length and the position of an entry hole. In our study, the central anatomical axis was significantly closer to that of the MA of the tibia compared to PAA (*p* < 0.01). Hence, CAA appears to be the best anatomical reference for tibial cut.

**Fig. 3:**
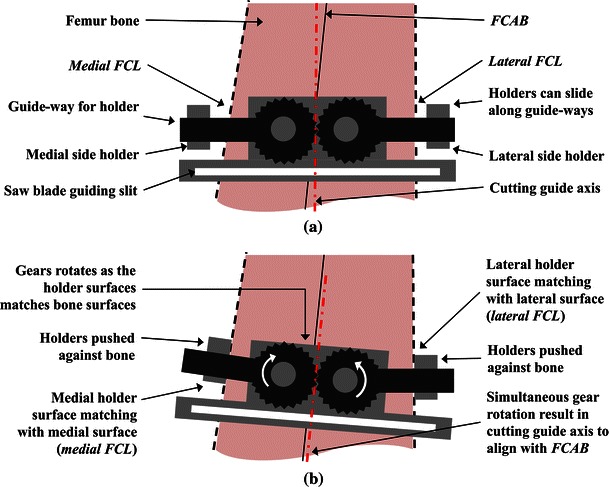
Schematics showing: **a** top view of a possible design concept of femoral cutting guide and **b** reference taken by the guide from both medial and lateral cortical surface simultaneously

The femoral study suggests that at distal region, none of the cortical surfaces can be used as anatomical reference. Hence, we also investigated the accuracy of using both the cortical surfaces simultaneously as indirect anatomical references. A new cutting guide can be designed that can use both medial and lateral cortical surfaces (FCLs) of femur as references, and give an alignment of their angle bisector using an appropriate mechanism. Femoral cortical angle bisector (FCAB) was defined as the line that bisects the angle between medial and lateral FCLs. The FCAB–FAA angle was directly calculated by subtracting the angle between lateral FCL and FAA from the angle between medial FCL and FAA, and dividing the difference by two. At the distal region, the mean value of the FCAB–FAA angle was 0.7° and at the central region, it was 0.1° (Table 4). We suggest that the FCAB gives the alignment of the anatomical axis, and hence the ideal cutting plane, more accurately than the individual FCLs, in the coronal plane. Figure 3 shows the top view of a possible design concept of a femoral cutting guide that holds the femur bone from both lateral and medial cortical surfaces (FCLs). The rotating gear mechanism allows the holders to rotate by equal angles simultaneously. As the two holders are pushed against cortical surfaces, the simultaneous gear rotation allows the cutting guide axis (which is perpendicular to the saw blade guiding slit) to get aligned with the angle bisector FCAB.

**Table 4:** Measurements of angles between the suggested anatomical references and axis perpendicular to the ideal cutting plane with their advantages

	Femur	Tibia
Suggested anatomical reference	Angle bisector of lateral and medial cortical lines (FCAB)	Central part of tibial canal accessed through an entry point at the knee center
Mean angle between anatomical reference and axis perpendicular to ideal cutting plane	Distal region: 0.7° (SD = 1.4) Central region: 0.1° (SD = 1.2)	0.5° (SD = 0.6)
Advantage	Most accurate at both distal and central region	No need of preoperative plan to determine the entry hole

**Fig. 4:**
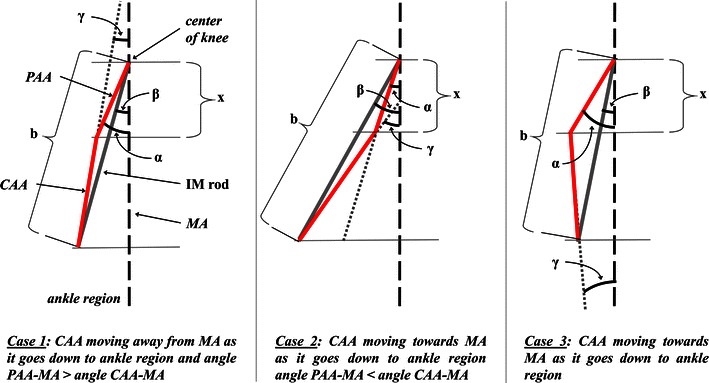
Schematic showing three possible cases of alignment of CAA with respect to MA

The tibial study suggests that a longer intramedullary rod parallel to CAA will give the alignment of ideal tibial cut more accurately. However, in that case, the entry hole will lie at the extension of CAA over tibial plateau. It usually lies over the anterior–lateral to the center of the knee [10], which can only be determined using preoperative X-rays. A patient-specific preoperative plan will, however, require considerable amount of time and effort. In the case where a surgeon uses a longer intramedullary rod but makes an entry hole at the center of knee joint (which can be easily identified without any preoperative plan), some angle (*β*) will lie between the rod and MA in the coronal plane, as shown in Fig. 4. There can be three possible cases according to the alignment of the CAA with respect to the MA (Fig. 4). By assuming the length of the intramedullary rod as 18 cm (7 inch) and using the values of the angles PAA–MA and CAA–MA found in this study, the angle ‘*β*’ can be calculated using Eqs. 1 and 2, derived for the three possible cases.

1 $$\text{Case}\;1\;\text{and}\;2:\beta =\gamma \;+\sin^{-1}\left\{\left(x/b\right)*\sin\left(\alpha -\gamma \right)/\cos\;\left(\alpha \right)\right\}$$

2 $$\text{Case}\;3:\beta =-\gamma +\sin^{-1}\left\{\left(x/b\right)*\sin\left(\alpha +\gamma \right)/\cos\left(\alpha \right)\right\}$$

where

*x* = 10 cm (in this study)

*b* = length of intramedullary (IM) rod

*α* = angle PAA–MA

*β* = angle between IM rod and mechanical axis (MA)

*γ* = angle CAA–MA

**Fig. 5:**
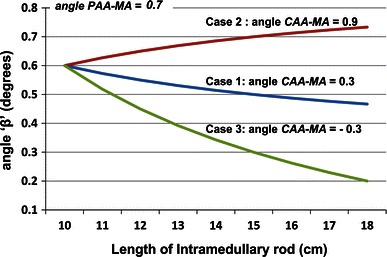
Variation of ‘*β*’ (angle between intramedullary rod and MA) with length of the intramedullary rod, for cases 1, 2 and 3

The mean value of the angle ‘*β*’ was found to be 0.5° with a standard deviation of 0.6° (Table 4). Hence, we suggest that the central part of the tibial canal, accessed through an entry hole at the knee center by a longer intramedullary rod, can be accepted as the best anatomical reference for knee replacements. This will eliminate the need for any preoperative plan to find an entry hole, required when using CAA as a reference, as described earlier. While using the knee center as an entry point, different lengths of intramedullary rod will give different values of ‘*β*’, depending on the case. Figure 5 shows the variation of angle ‘*β*’ with the length of intramedullary rod. For constant values of PAA–MA and CAA–MA angles (depending on the case), the angle ‘*β*’ decreases rapidly in case 3, but increases rapidly in case 2, as the length of the intramedullary rod increases. Since case 2 occurs rarely (12 % of all the cases in the tibial study), a longer intramedullary rod will give the alignment of the MA, and hence the ideal tibial cut more accurately.

The measurements were performed in the scanograms of patients of Indian origin, and hence the results may not be applicable to a different ethnicity. Another limitation is that the study was performed using two-dimensional (2D) X-rays where the rotational positioning of the implant component cannot be studied. Berhouet et al. had presented a study based on three-dimensional (3D) computed tomography (CT) scans, where using the rotational alignment of femoral component as a reference for the rotational alignment of the tibial component was analyzed [2]. The 3D imaging and representation enables viewing the anatomy more accurately and realistically. Intraoperative navigation based on 3D bone models are hence generally assumed to be superior to the conventional surgical guides [1, 17]. Recent studies, however, show that there is no difference in clinical function, alignment and survivorship of the components between the knees that underwent computer-navigated surgeries and those that underwent conventional surgeries [9, 16]. The conventional instruments are accurate from an engineering point of view, and should be improved in terms of how are they employed [15]. Our study is an attempt to improve the performance of the conventional instruments by using the selected best anatomical references.

In conclusion, for a femoral cut, both lateral and medial cortical surfaces can be used as the anatomical references, but only at the central region. For both central and distal femoral cuts, the bisector of angle between the cortical lines is nearly parallel to the anatomical axis, which suggests designing a new cutting guide that can determine the angle bisector of medial and lateral cortical lines by using them as indirect references. For the tibia, the central anatomical axis can be used as a reference to get an accurate tibial cut. The central part of the tibial canal accessed through an entry hole at the knee center is also suggested as a reference in the absence of preoperative plan.
